# *Bacillus subtilis* Modulated the Expression of Osteogenic Markers in a Human Osteoblast Cell Line

**DOI:** 10.3390/cells12030364

**Published:** 2023-01-19

**Authors:** Jerry Maria Sojan, Caterina Licini, Fabio Marcheggiani, Oliana Carnevali, Luca Tiano, Monica Mattioli-Belmonte, Francesca Maradonna

**Affiliations:** 1 Department of Life and Environmental Sciences, Università Politecnica delle Marche, Via Brecce Bianche, 60131 Ancona, Italy; 2 Department of Clinical and Molecular Sciences (DISCLIMO), Università Politecnica delle Marche, Via Tronto 10/a, 60126 Ancona, Italy

**Keywords:** ossification genes, probiotics, hFOB 1.19 cells, vitamin D, osteoblasts, mineralization, alkaline phosphatase

## Abstract

Several in vivo trials have previously demonstrated the beneficial effects of the administration of various probiotic forms on bone health. In this study, we explored the potency of two probiotics, *Bacillus subtilis* and *Lactococcus lactis*, alone or in combination with vitamin D (VD), to modulate the transcription of genes involved in the ossification process in a human osteoblast cell line. Genes that mark the “osteoblast proliferation phase”, such as *RUNX2*, *TGFB1*, and *ALPL*, “extracellular matrix (ECM) maturation”, such as *SPP1* and *SPARC*, as well as “ECM mineralization”, such as *BGN*, *BGLAP*, and *DCN*, were all highly expressed in osteoblasts treated with *B. subtilis* extract. The observed increase in the transcription of the *ALPL* mRNA was further in agreement with its protein levels as observed by Western blot and immunofluorescence. Therefore, this higher transcription and translation of alkaline phosphatase in osteoblasts treated with the *B. subtilis* extract, indicated its substantial osteogenic impact on human osteoblasts. Although both the probiotic extracts showed no osteogenic synergy with VD, treatment with *B. subtilis* alone could increase the ECM mineralization, outperforming the effects of *L. lactis* and even VD. Furthermore, these results supported the validity of employing probiotic extracts rather than live cells to investigate the effects of probiotics in the in vitro systems.

## 1. Introduction

Bone continuously undergoes formation by osteoblasts as well as resorption by osteoclasts and every disruption to this fine balance can lead to unfavorable bone health conditions, such as osteoporosis, which is a vast health issue [1,2]. The increase and differentiation of osteoblasts can be broadly categorized into three distinct phases: Proliferation, extracellular matrix (ECM) production and maturation and mineralization. Several molecular markers were previously identified to have a distinct upregulated expression during these phases. The key-regulator RUNX family transcription factor 2 (*RUNX2*), which is highly expressed in the proliferation stage, also regulates the transcription of other genes related to ECM maturation and mineralization phases, such as alkaline phosphatase, biomineralization associated (*ALPL*), and bone gamma-carboxyglutamate protein (*BGLAP*) [3]. *RUNX2* regulation of osteogenesis can be carried out through many possible ways as this gene is part of several bone-related signaling pathways, including transforming growth factor-β (TGF-β), bone morphogenetic protein (BMP), and fibroblast growth factor (FGF) [4]. For the process of mineralization of the ECM to start, alkaline phosphatase (ALP) activity must be higher since phosphate ions are required for mineral deposition [5]. The upregulation of specific gene expression during these three phases [6,7,8] (summarized in Table 1) can be useful to study the phase-effects of any treatments on the osteoblasts.

Probiotics are beneficial microbes that have numerous positive effects on the host if administered in suitable quantities. There are previous reports describing the positive role of some probiotic species in improving bone parameters by increasing bone mass density or decreasing bone loss [9,10,11,12] and accelerating zebrafish caudal fin bone regeneration [13]. To date, limited studies have reported the effects of probiotics on bone cells in vitro since they are live microbes that can easily contaminate the cell cultures if directly used in the cultures. A previous study reported an increase in osteoblastic activity in mouse cell cultures supplemented with milk fermented using *Lactobacillus helveticus* [14]. There have been only a few reports of researchers investigating the various beneficial effect of probiotics in cell cultures using probiotic extracts, supernatants, or even fractions of probiotic cultures [15,16,17,18].

Therefore, the present study aimed at establishing a suitable in vitro method to check the efficiency of probiotics in modulating osteoblast differentiation and inducing mineralization of ECM. Moreover, we aimed to explore the possibility of bacteria producing vitamin K2 (menaquinones) in promoting a synergistic effect with vitamin D (VD) on the ossification process. Ethanol extracts of two probiotics were used alone and in combination with an established osteogenic concentration of VD to treat hFOB1.19 cells. This cell line is an ideal model to study in vitro bone formation, capable of generating ECM with ultrastructural elements similar to those deposited by primary osteoblasts [19]. Cell survival, ALP production, and calcium nodule formation were analyzed in all experimental groups. Osteoblasts are responsible for the expression of ALP, an important enzyme in the process of biomineralization in bone, and this protein may be found tethered to the cell membrane or matrix vesicles [20,21]. To further understand the molecular players involved in various phases of osteoblast differentiation modulated by the probiotic extracts, the expression of selected marker genes was analyzed. In addition, Western blotting (WB) and immunofluorescence techniques were employed to validate the subsequent translated protein levels of the selected genes.

## 2. Materials and Methods

### 2.1. Cell Culture and Treatments

Human fetal hFOB 1.19 SV40 large T antigen transfected osteoblastic cells (ATCC CRL-11372) were cultured in 1:1 mixture of Ham’s F12 Medium and Dulbecco’s Modified Eagle’s Medium (DMEM) with 2.5 mM L-glutamine and without phenol red (Life Technologies Limited, Paisley, UK), supplemented with 0.3 mg/mL of G418/Geneticin (Life Technologies Corporation, Grand Island, NY, USA) and 10% Fetal Bovine Serum (FBS; Life Technologies Limited, Paisley, UK) at 34 °C and 5% CO_2_. For the experiments, the cells were seeded in an osteogenic media comprising 50 μg/mL ascorbic acid (Sigma-Aldrich, St. Louis, MO, USA) and 7.5 mM β-glycerophosphate (Sigma-Aldrich, St. Louis, MO, USA) to stimulate mineralization for 7 days [22,23]. Cells were seeded at a density of 1 × 10^5^ cells/well in 12-well plates. After seeding, the medium was removed from each well on the second day when the cells were attached to the bottom of the wells, and media with corresponding treatments were added to the monolayer of cells (1 mL/well). Media with respective treatments were changed on the fourth day. VD (calcitriol or vitamin D3; Sigma-Aldrich, Milan, Italy) and high-performance liquid chromatography (HPLC) ethanol extracts of two probiotics—*Bacillus subtilis* (P1) and *Lactococcus lactis* (P2) (both from Fermedics, Ghent, Belgium) were used for creating the treatment groups. Probiotics were tested alone or in combination with VD for a total of 6 treatment groups as shown in Table 2.

### 2.2. HPLC Quantification of Vitamin K2 or Menaquinones (MK) from Probiotics

Using HPLC, MK-7 and MK-9 were measured to confirm the production ability of MKs by the bacteria. A total of 50 mg of bacteria was extracted with 1 mL of ethanol and vortexed vigorously for at least 30 s. Subsequently, to maximize the MKs extraction, 3 cycles of 3 min each of sonication interspersed with 30 s of vortex were performed. The suspension was centrifuged at 20,900× *g* for 2 min at 4 °C and 40 µL of supernatant was injected in the HPLC system (9300, YL Instrument, Anyang, Republic of Korea) and the two MK forms were quantified using fluorescence detector (Nanospace SI-2, Shiseido, Tokyo, Japan). K vitamin levels were assayed in the ethanol extract using an analytical column (2.6 µm C18 100A, 100 × 4.6 mm; Phenomenex Kinetex, Torrance, CA, USA) connected to a post-chromatographic reducing column (CQ-R 2.0 × 20 mm; Shiseido, Tokyo, Japan). The mobile phase used was ethanol: water (97:3, *v*/*v*) and the flow rate was adjusted to 0.7 mL/min. The optimized detection wavelengths were 335 nm (excitation) and 430 nm (emission). Under these conditions, the detection was 10 min long and it was possible to separate MK-7 and MK-9. External standards (purchased from Sigma-Aldrich, St. Louis, MO, USA) were used to quantify K vitamin concentrations. Results are expressed as µg/g for both MK-7 and MK-9.

### 2.3. XTT Assay

The hFOB1.19 cell viability under different treatments for three time points—1, 2, and 7 days was determined using the XTT Assay Kit (ab232856, Abcam, Cambridge, UK) according to the manufacturer’s instructions. Cells were seeded in 96-well plates (*n* = 5) at a density of 10^4^ cells/well in 100 µL of culture medium with all the experimental groups to be tested and incubated at 37 °C and 5% CO_2_. A total of 10 µL of the prepared XTT mixture (equal volumes of XTT developer reagent and electron mediator solution) were added to each well after 1, 2, and 7 days and incubated for 2 h at 37 °C and 5% CO_2_. After incubation, absorbance was measured using a microplate reader at 450 nm.

### 2.4. Alizarin Red (AR) Staining

After 7 days of culture in 12-well plates with respective treatments (*n* = 5) in triplicates, the cells were washed 3 times with PBS and fixed in 4% (*v*/*v*) of PFA (1 mL/well) for 30 min at 4 °C. The PFA was discarded and the cells were then washed 3 times with milli-Q water. The fixed cells were stained with 40 mM AR (pH 4.2, Fluka Chemika, Buchs, Switzerland) for 30 min in the dark at room temperature. Finally, monolayers were washed with milli-Q water 4 times and were observed under Lionheart XF Automated Microscope (Biotek, Winooski, VT, USA). Calcium nodules appeared in red. For the spectroscopic quantification of calcium deposits, the distilled water was removed and the AR stain was dissolved in 1 mL of 10% cetylpyridinium chloride (CPC) (Sigma-Aldrich, St. Louis, MO, USA) for 30 min at room temperature. Finally, 250 µL of the solution was obtained from each well and then transferred to a 96-well plate to measure absorbance at 550 nm.

### 2.5. ALP Staining

Cells were fixed and washed using the same protocol as the AR staining described in the previous section and the fixed cells were incubated with a staining mixture for ALP provided in the BCIP/NBT kit (Sigma-Aldrich, St. Louis, MO, USA) for 45 min at 37 °C in dark conditions following the kit protocol. Once the color developed, the dye was removed and stained monolayers were washed 2 times and covered with milli-Q water (1 mL/well) and photographed using a Lionheart XF Automated Microscope (Biotek, Winooski, VT, USA).

### 2.6. WB

The following proteins—ALP, Osteonectin (ON), Osteopontin (OPN), and Osteocalcin (OC) were quantified by WB in hFOB1.19 cells after 7 days of probiotic treatments. The contents of 3 wells for each treatment group were pooled in 1.5 mL Eppendorf and two biological replicates per group were sampled for protein extraction. Each WB was repeated 4 times. Total proteins were extracted using radio-immunoprecipitation assay buffer system (RIPA Lysis Buffer System, Santa Cruz Biotechnology, Dallas, TX, USA). Protein concentration was determined using DC protein assay kit (BIO-RAD, Hercules, CA, USA) by reading absorbance at 750 nm. Total protein extracts (15 µg) were incubated with NuPAGE™ LDS Sample Buffer (4X) (Invitrogen, Waltham, MA, USA) according to the manufacturer’s instructions, fractionated in NuPAGE™ 4–12% Bis-Tris Protein Gels (Invitrogen, Waltham, MA, USA) and electrophoretically transferred to PVDF membranes. Membranes were incubated with 5% milk in Tris-buffered saline (TBS-T) with 0.1% Tween 20 to block non-specific sites or 5% bovine serum albumin (BSA) in TBS-T for OPN (since OPN is a phosphoprotein and milk blockage can cause background) and then with anti-ON (35 and 45 kDa fragments) (dilution 1:250, Santa Cruz Biotechnology, Dallas, TX, USA), anti-ALP (78 and 200 kDa fragments) (dilution 1:1000, Abcam, Cambridge, UK), anti-OPN (dilution 1:250, Santa Cruz Biotechnology, Dallas, TX, USA), and anti-OC (dilution 1:250, Santa Cruz Biotechnology, Dallas, TX, USA) primary antibodies at 4 °C. Mouse anti-GAPDH (dilution 1:10,000, Proteintech group, Rosemont, IL, USA) was used as an endogenous control. After overnight incubation, the membrane was washed 4 times with TBS-T and then incubated for 90 min at room temperature with the secondary antibodies conjugated to horseradish peroxidase and then washed 4 times with TBS-T. The detection of antibody binding was performed with Pierce ECL WB Substrate (Thermo Scientific, Waltham, MA, USA) and images were taken using Alliance Mini HD9 (Uvitec, Cambridge, UK).

### 2.7. Immunofluorescence Staining

For immunofluorescence staining, cells were fixed in 4% PFA for 30 min at room temperature and washed 3 times with phosphate-buffered saline (PBS). Then, cells were permeabilized using 0.1% Triton X-100 in PBS 1X for 30 min and washed 3 times with PBS followed by 30 min of blocking with 1% BSA in PBS. Thereafter, cells were incubated overnight with mouse anti-human ALP primary antibody (dilution 1:100, Abcam, Cambridge, UK) (green fluorescence) at 4 °C. Next, cells were first washed 3 times with PBS and then stained with a FITC-conjugated secondary antibody (dilution 1:1500, Invitrogen, Waltham, MA, USA) for 30 min protected from light. After washing 3 times with PBS, TRITC-labelled phalloidin (1:100, Invitrogen, Waltham, MA, USA) diluted in PBS was added to visualize F-actin fiber organization (red fluorescence) for 45 min and washed 3 times with PBS. Nuclei were counterstained (blue fluorescence) with DAPI (1:1000) for 10 min and washed 3 times with PBS. Samples were mounted using Vectashield mounting medium (Vector Laboratories Inc, Newark, CA, USA) and observed with a Nikon E600 Fluorescence microscope (Nikon, Milan, Italy). Semi-quantitative analysis was performed to calculate the corrected total cell fluorescence (CTCF).

### 2.8. Image Analysis

ImageJ version 2.1.0/1.53c software was used for the image analysis of AR staining, ALP staining, WB bands, and immunofluorescence staining.

### 2.9. RNA Extraction and Quantification

Cells were cultured in 12-well plates as previously described. After 7 days of culture, the cells were detached and lysed directly into plates by removing the media and adding 100 μL of RNAzol to each well (vigorously pipetting to detach all adherent cells). The contents of 3 wells for each treatment group were pooled in 1.5 mL Eppendorf and four biological replicates per group, each from a different plate, were sampled for RNA extraction. Then, total RNA was extracted from the cells using RNAeasy Minikit (Qiagen, Milan, Italy) following the manufacturer’s instructions. Thereafter, it was eluted in 20 µL of molecular grade nuclease-free water. Final RNA concentrations were determined using a nanophotometer (Implen GmbH, Munich, Germany). Total RNA was treated with DNase (10 IU at 37 °C for 10 min, MBI Fermentas, Milan, Italy). Then, 1 µg of total RNA was used for cDNA synthesis using iScript cDNA Synthesis Kit (Bio-Rad, Milan, Italy) according to the manufacturer’s instructions and stored at −20 °C until further use.

### 2.10. Quantitative Reverse Transcription Polymerase Chain Reaction (qRT-PCR)

qRT-PCRs were performed in triplicate on 4 biological replicate samples per treatment with SYBR green (Bio-Rad, Milan, Italy) in a CFX thermal cycler (Bio-Rad, Milan, Italy) as previously described [13]. The thermal profile for all reactions was 3 min at 95 °C followed by 45 cycles of 20 s at 95 °C, 20 s at 60 °C, and 20 s at 72 °C. Dissociation curve analysis showed a single peak in all the cases. Actin beta (*ACTB*) and ribosomal protein lateral stalk subunit P0 (*RPLP0*) were used as the housekeeping genes to standardize the results by eliminating variation in mRNA and cDNA quantity. Data were analyzed using iQ5 Optical System version 2.1 (Bio-Rad, Milan, Italy) including Genex Macro iQ5 Conversion and Genex Macro iQ5 files. Modification of gene expression levels between the experimental groups is reported as relative mRNA abundance (Arbitrary Units). Primers are used at a final concentration of 10 pmol/mL. All primer sequences used in the study are listed in Table 3.

### 2.11. Statistical Analysis

Data of all groups were normally distributed, as assessed by Shapiro-Wilk’s test (*p* > 0.05) and variances were homogeneous, as assessed by Levene’s test for equality of variances (*p* > 0.05). The differences between the control and the treatments were tested with a two-way analysis of variance (ANOVA) for the XTT assay data and one-way ANOVA for all other data, followed by Tukey’s post hoc test. The significance level is set at a constant *p* < 0.05 in all the cases but the focus was on all possible pair-wise comparisons. All the tests were performed using R (version 3.6.1) [24] and plots were generated using ggplot2 (version 3.2.1).

## 3. Results

### 3.1. MKs Production

The average production of two forms of MKs, MK-7 and MK-9, from the two probiotic bacteria was quantified respectively as:

▪ P1: 81 and 0.5 µg/g;
▪ P2: 0.59 and 7 µg/g.

Furthermore, the unquantified peaks observed in the HPLC output could be other forms of MKs produced by the bacteria, such as MK-8 (Supplementary Figure S1).

### 3.2. Cell Viability

**Figure 1:**
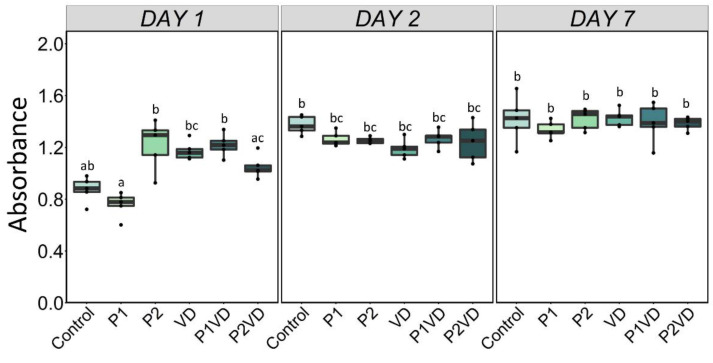
Cell viability analyzed by XTT assay in human fetal osteoblasts (hFOB1.19) exposed to different treatments—cultured without any treatment (Control), treated with VD (VD), with two probiotics (P1 and P2) and their respective combinations with VD (P1VD and P2VD). Two-way ANOVA was used to analyze the difference among the groups and Tukey’s post hoc test was used for multiple comparisons among every group. Data are shown as means ± SD as error bars. Different letters above each column denote statistically significant differences among all experimental groups after 1, 2, and 7 days (*n* = 5, *p* < 0.05). Three groups that differ significantly from each other are labelled a, b, and c, and two that differ from each other but not from the third are labelled a, b, and ab.

The XTT test was used to determine cell proliferation and viability in all treatment groups after 1, 2, and 7 days. After 1 day, no significant change was found between control and all the other groups. However, we found a significant decrease in cell number in the P1 group relative to the P2, VD, and P1VD groups. However, after 2 days, all treatments showed a similar number of viable cells with respect to the control (Figure 1).

### 3.3. Calcium Nodules and ALP Production

**Figure 2:**
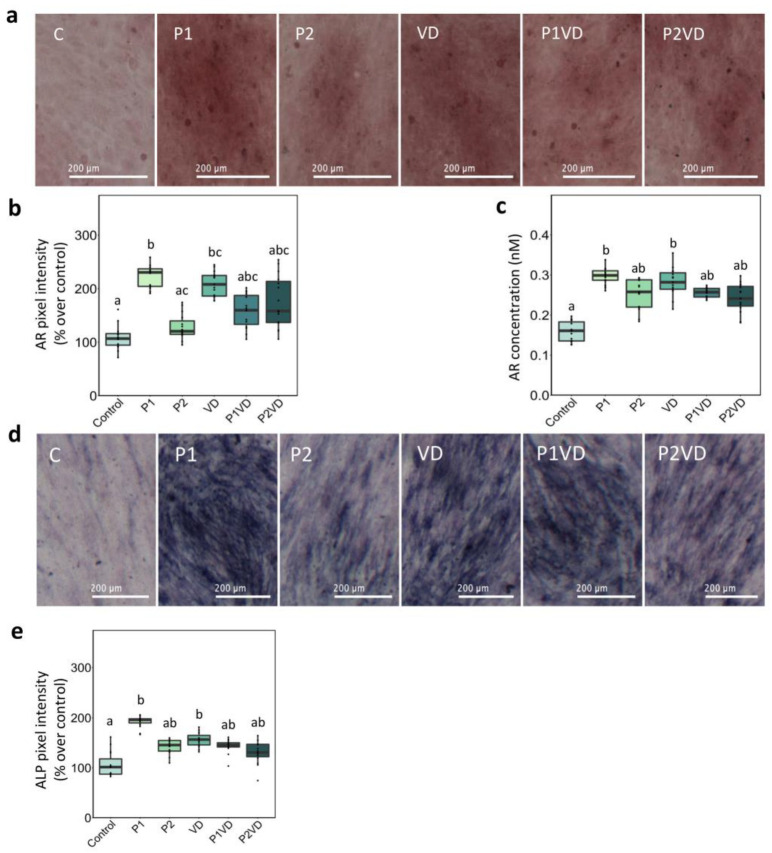
Staining of the hFOB1.19 cells cultured in the different treatments—cultured without any treatment (Control), treated with VD (VD), with two probiotics (P1 and P2) and their respective combinations with VD (P1VD and P2VD): (**a**) Representative images of the different treatments applied to hFOB1.19 cells for 7 days after AR staining, Scale bar: 200 µm; (**b**) AR pixel intensity measured using ImageJ and converted to % values over control; (**c**) Quantitative analysis of the AR concentration indicative of the ECM mineralization following the different treatments by measuring the absorbance using spectroscopy; (**d**) Representative images of the different treatments applied to hFOB1.19 cells for 7 days after ALP staining, Scale bar: 200 µm; (**e**) ALP pixel intensity measured using ImageJ and converted to % values over control. The graphs show the mean and standard deviation as error bars. One-way ANOVA was used to analyze the difference between the groups and Tukey’s post hoc test was used for multiple comparisons among groups. Data are shown as means ± SD as error bars. Different letters above each column denote statistically significant differences among experimental groups (*n* = 5, *p* < 0.05). Three groups that differ significantly from each other are labelled a, b, and c, and two that differ from each other but not from the third are labelled a, b, and ab.

AR staining was used to compare the capacity of the hFOB1.19 cells to produce calcium nodules after the treatments. As expected, there was a significant difference between the control and the VD, where VD treated cells exhibited more mineralized nodules, as qualitatively indicated by the more prominent red color in the images and quantitatively by the estimation of the image pixel intensity. Moreover, spectrometric analysis demonstrated that VD treated cells had higher AR concentration than the control (Figure 2a–c). It is important to note that a considerable rise in the occurrence of calcium nodules, similar to VD group, was also observed in the group that had been treated with the P1 extract (Figure 2a–c).

ALP cytochemical staining of the hFOB1.19 cells was used to quantify the osteogenic differentiation in the cells. Both P1 and VD induced a significant increase in ALP activity, as seen in the images, which was further confirmed by comparing the pixel intensities (Figure 2d,e).

### 3.4. Protein Expression

**Figure 3:**
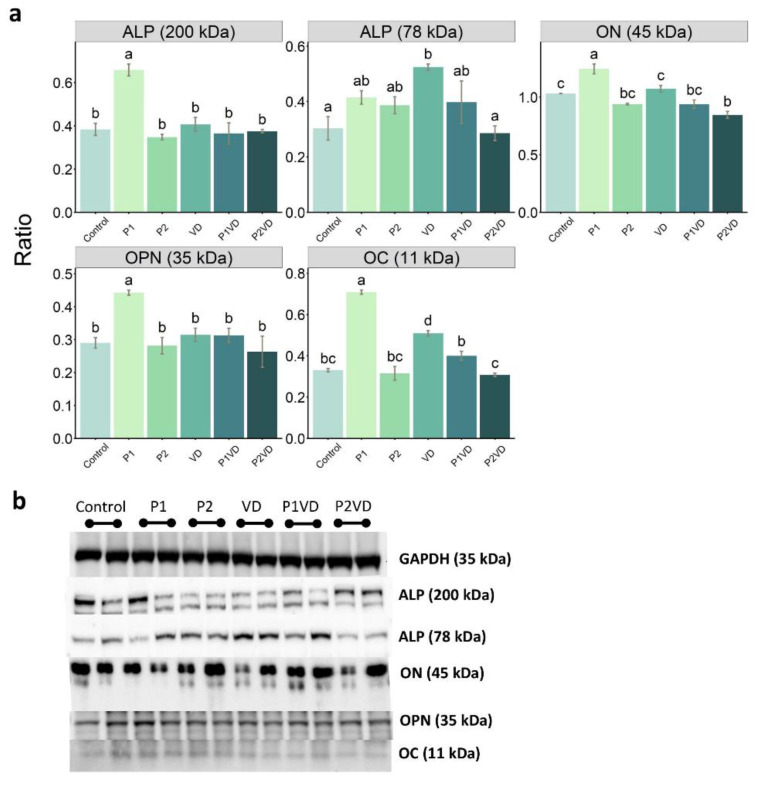
WB analysis of the expression of bone formation related proteins: ALP (200 and 78 kDa), ON, OPN, and OC in hFOB1.19 cells cultured without any treatment (Control), treated with VD (VD), with two probiotics (P1 and P2) and their respective combinations with VD (P1VD and P2VD): (**a**) Relative expression of the proteins calculated as a ratio of band density with respect to glyceraldehyde 3-phosphate dehydrogenase (GAPDH). One-way ANOVA was used to compare between groups and Tukey’s post hoc test was used for multiple comparisons among all the groups. Data are shown as means ± SD as error bars. Different letters above each column denote statistically significant differences among experimental groups (*n* = 2, *p* < 0.05). Three groups that differ significantly from each other are labelled a, b, and c, and two that differ from each other but not from the third are labelled a, b, and ab; (**b**) Representative WBs (*n* = 4) visualized by enhanced chemiluminescence method for GAPDH, ALP (200 and 78 kDa), ON, OPN, and OC proteins.

The increase in mineralized nodules and ALP activity observed in the P1 group was further validated by performing WB analysis for selected proteins including ALP (Figure 3a,b). OC and the 200 kDa form of ALP were significantly more abundant in the P1 samples with respect to the control. In addition, both ON and the 78 kDa form of ALP were found to be significantly lowered in P2VD cells when compared to VD suggesting a negative action of P2 extract when combined with VD. Regarding OPN, a significant increase in P1 cells was found and interestingly no change in OPN was detected in the cells exposed to VD with respect to the control (Figure 3a).

### 3.5. ALP Immunofluorescence

**Figure 4:**
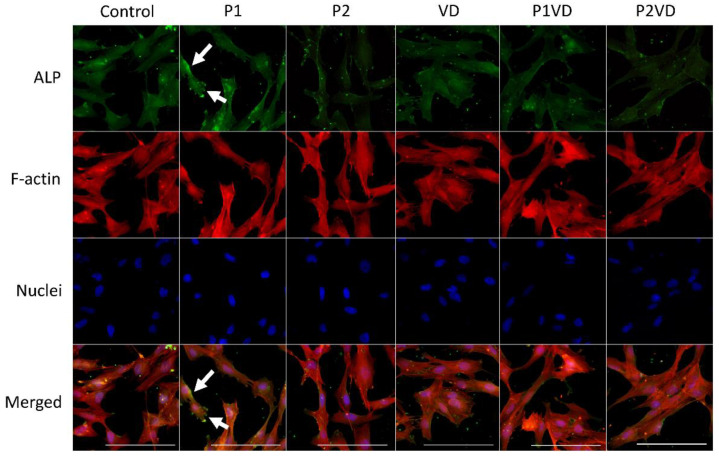
Representative immunofluorescence detection images of ALP, F-actin cytoskeleton (Phalloidin), and nuclei (DAPI) in hFOB1.19 cells cultured without any treatment (Control), with VD (VD), two probiotics (P1 and P2) and their respective combinations with VD (P1VD and P2VD). White arrows indicate the highest immunofluorescence of ALP detected in P1 cells; Scale bar: 100 µm.

ALP fluorescent staining, along with staining of cytoskeletal filamentous actin fibers and nuclei, was performed on the osteoblasts after 7 days of culture with corresponding treatments (Figure 4). After 1 week of treatment, the cells of all the groups exhibited a characteristic adherent morphology with a dispersion of well-structured F-actin filaments. ALP, a protein involved in mineralization, was detected more in the cells treated with P1 extracts. ALP was particularly localized throughout the inner surface of the cell membrane which was observed as brighter green spots along the membranes (Figure 4, white arrows). Interestingly, ALP staining was observed in correspondence with rounded structures near the cell membrane, resembling the usual disposition of the protein in matrix vesicles. Semi-quantitative analysis of the fluorescence further validates that P1 treated cells have a higher mean total cell fluorescence for ALP than control cells (Supplementary Figure S2).

### 3.6. Gene Expression

**Figure 5:**
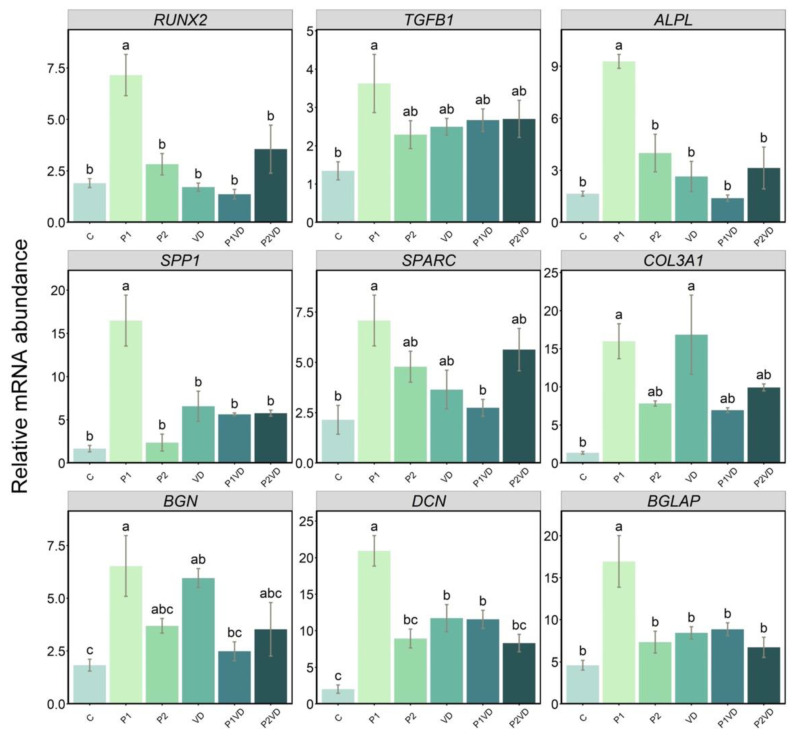
Expression of genes involved in the osteoblast proliferation, maturation, and mineralization of the ECM, in cells cultured without any treatment (C), treated with VD (VD), with two probiotics (P1 and P2) and their respective combinations with VD (P1VD and P2VD). Data are shown as means ± SD as error bars. One-way ANOVA was used to analyze the difference between the groups and Tukey’s post hoc test was used for multiple comparisons among groups. Different letters above each column denote statistically significant differences among experimental groups (*n* = 4, *p* < 0.05). Three groups that differ significantly from each other are labelled a, b, and c, and two that differ from each other but not from the third are labelled a, b, and ab.

The expression of selected marker genes of various stages of osteoblast growth and differentiation was evaluated after the treatments (Figure 5). Regarding genes involved in the “proliferation stage” and *RUNX2*, transforming growth factor beta 1 (*TGFB1*) mRNAs were significantly highly expressed in P1 extract-treated cells. Neither of the synergy groups nor VD was showing any significant difference in the mRNA levels of these genes. Regarding the “maturation of ECM” stage marker genes *ALPL*, secreted phosphoprotein 1 (*SPP1*), secreted protein acidic and rich in cysteine (*SPARC*), and collagen type III alpha 1 chain (*COL3A1*) mRNAs showed a significant increased expression in the P1 group. “Mineralization of ECM” stage marker genes biglycan (*BGN*), *BGLAP*, and decorin (*DCN*) were also found to be highly expressed in the P1 group. Moreover, the VD group exhibited a higher expression for genes involved with the “maturation of ECM”, such as *COL3A1* and for the mineralization markers, *DCN* and *BGN*, compared to the control. Interestingly, in P1VD, *DCN* was significantly higher than the control (Figure 5).

## 4. Discussion

Compounds with an anabolic impact on bone may be useful in enhancing the activity of osteoblasts and for treating osteoporosis, which is a degenerative condition of aged bone and a major public health concern on a global scale due to its high prevalence [2]. During bone formation, osteoblasts undergo a series of distinct phases of differentiation and maturation which involves stages, such as proliferation, matrix synthesis, and mineralization [25]. To date, more studies are required to determine which substances may favorably affect these processes. A great deal of interest has been shown in prophylactic medications or nutritional supplements originating from natural sources, such as probiotics. In agreement with previous studies [26], no harmful effect was shown by VD on cell proliferation at the selected concentration in this study. Most importantly, we could show that the use of ethanol extract from the two probiotics did not cause any morphological changes to the cells or damage to the cytoskeleton; therefore, it could be proposed as a suitable method to study the in vitro effects of probiotic species.

The two groups that could enhance the amount of ALP produced were the extract of *B. subtilis* and VD. *B. subtilis* species is a well-known producer of MK forms, such as MK-7 [27]. Additionally, the MKs were previously reported to enhance the osteogenic effect in mouse osteoblasts by significantly increasing the activity of ALP [28]. Moreover, we quantified the two forms of MKs in the probiotic extracts by HPLC and the confirmed quantities of MKs, mainly MK-7, could be the reason for the enhanced ALP production seen with *B. subtilis* extract. This was further validated by immunofluorescence and WB on the osteoblasts treated with *B. subtilis*. ALP was found to accumulate in the inner surface of the cell membrane and structures resembling matrix vesicles, notably involved in the mineralization process. Furthermore, WB revealed that functional ALP (200 kDa) and OC levels were significantly higher in the *B. subtilis* treated cells. The presence of ALP activity, which is a phenotypic marker for osteoblasts appearing during the differentiation process, and the mineralized nodules evidenced by AR, occurring later in the differentiation process, indicate that *B. subtilis* extracts were able to promote differentiation of osteoblasts. Higher calcium deposition observed in the cells treated with *B. subtilis* extracts could be accounted to the *B. subtilis*’s superior MK producing capacity since MKs were previously found to promote the formation of extracellular mineralized nodules in MC3T3-E1 cells [29]. In addition, the increased *RUNX2* and *TGFB1* expression provided insight into the influence of probiotic extract fractions on osteoblasts proliferation. *RUNX2* is essential for proper skeletal development since it controls chondrocyte and osteoblast differentiation [3]. Several bone matrix protein genes, such as *SPP1* and *BGLAP*, are upregulated by *RUNX2* and their respective promoters are also activated in vitro by this transcription factor [3]. *ALPL* gene expression, which is also known to be regulated by *RUNX2*, is a critical marker for osteoblast maturation and the ALP protein can control the mineralization process by creating free inorganic phosphates [30,31]. The accumulation of these inorganic phosphates and calcium ions within matrix vesicles is presumed to result in the formation of amorphous calcium phosphate or hydroxyapatite crystals, which are considered to be the first stage of ECM mineralization during the process of osteogenesis [32]. *B. subtilis* extracts upregulated the master osteogenesis regulator gene *RUNX2* in a favorable manner, which is further associated with the elevated expression of *ALPL* and *SPP1* genes, both of which are targets of this transcription factor. *TGFB1*, another factor involved in ECM deposition, plays a crucial function in the remodeling of bone [7,33], which is also found to be elevated in the *B. subtilis* treated cells. Moreover, in our study, we noticed a uniform upregulation of *SPP1*, *COL3A1*, *BGLAP*, and *DCN* in all groups during the last phases of osteoblast differentiation, which includes the maturation and mineralization of the ECM, and this is indicative of the higher differentiation status of the cells grown with *B. subtilis* extracts.

These overall findings, along with MK’s very low toxicity, may be attributable to the exceptional osteogenic performance shown by *B. subtilis* in this study. This same probiotic *B. subtilis* was able to exert its osteogenic effect in vivo in zebrafish transgenic larvae as observed in one of our previous studies [34]. MKs have been therapeutically used to prevent osteoporosis, and they are thought to exert protective effects via increasing osteoblast development and mineralization in the bone matrix [35,36]. There are previous reports of the capability of MKs in increasing the expression of *ALPL*, *RUNX2*, and several ECM related genes as well as collagen accumulation in addition to promoting osteoblast maturation in vitro [37,38,39,40,41,42]. The current study establishes for the first time, to our knowledge, that probiotic extracts can increase osteoblast differentiation and ECM mineralization in human osteoblasts, which may open new avenues for the investigation and development of functional foods for promoting bone formation to prevent osteoporosis. However, the complete molecular mechanisms behind probiotic extract’s osteogenic action, such as finding out whether these probiotic extracts influence the cellular start of mineralization through the direct activation of promoters and repressors or by some indirect influence, requires additional exploration. More importantly, separation and characterization of the highly active fractions in addition to the MK forms are recommended for further molecular investigation.

## Figures and Tables

**Table 1:** Some of the bone related genes with the upregulation of expression during the three phases of osteoblast lineage cell growth and differentiation: Proliferation, ECM production and maturation, and ECM mineralization [6,7,8].

Proliferation	ECM Maturation	ECM Mineralization
*TGFB1*	*SPP1*	*ITGB1*
*RUNX2*	*BGLAP*	*ITGB3*
*RUNX3*	*SPARC*	*ITGB5*
*NELL1*	*ALPL*	*RUNX3*
*BMP7*	*IBSP*	*DCN*
*IBSP*	*COL3A1*	*COL3A1*
*COL1A1*		
*COL2A1*		
*ALPL*		

**Table 2:** Composition of all treatment groups.

Groups	Composition
Control (C)	DMEM + FBS 10% + 0.03 mg/mL G418
P1	DMEM + FBS 10% + 0.03 mg/mL G418 + P1 extract 1 µL/mL
P2	DMEM + FBS 10% + 0.03 mg/mL G418 + P2 extract 1 µL/mL
VD	DMEM + FBS 10% + 0.03 mg/mL G418 + 100 nM VD
P1VD	DMEM + FBS 10% + 0.03 mg/mL G418 + P1 extract 1 µL/mL + 100 nM VD
P2VD	DMEM + FBS 10% + 0.03 mg/mL G418 + P2 extract 1 µL/mL + 100 nM VD

**Table 3:** List of primers used in gene expression analysis by qRT-PCR.

Gene	Forward Primer (5′-3′)	Reverse Primer (3′-5′)
*ACTB*	ATTGGCAATGAGCGGTTC	GGATGCCACAGGACTCCAT
*RPLP0*	CTGGAAAACAACCCAGCTCT	GAGGTCCTCCTTGGTGAACA
*RUNX2*	GTGCCTAGGCGCATTTCA	GCTCTTCTTACTGAGAGTGGAAGG
*ALPL*	CCATCCTGTATGGCAATGG	CGCCTGGTAGTTGTTGTGAG
*TGFB1*	GCAGCACGTGGAGCTGTA	CAGCCGGTTGCTGAGGTA
*COL3A1*	CTGGACCCCAGGGTCTTC	CATCTGATCCAGGGTTTCCA
*SPP1*	TTGCAGCCTTCTCAGCCAA	CAAAAGCAAATCACTGCAATTCTC
*SPARC*	GTACATCGCCCTGGATGAGT	CGAAGGGGAGGGTTAAAGAG
*BGN*	CAGCCCGCCAACTAGTCA	GGCCAGCAGAGACACGAG
*DCN*	GGAGACTTTAAGAACCTGAAGAACC	CGTTCCAACTTCACCAAAGG
*BGLAP*	ACACTCCTCGCCCTATTG	GATGTGGTCAGCCAACTC

## Data Availability

Not applicable.

## Supplementary Materials

The following supporting information can be downloaded at: https://www.mdpi.com/article/10.3390/cells12030364/s1, Figure S1: MK-7 and MK-9 peaks of the standard and in the two probiotic species observed in the HPLC output; Figure S2: Semi-quantitative analysis to calculate corrected total cell fluorescence (CTCF) in hFOB1.19 14 cells cultured without any treatment (Control), treated with VD (VD), with two probiotics (P1 and P2) and their re-15 spective combinations with VD (P1VD and P2VD.
