# Supplementary material for: Bacillus subtilis Modulated the Expression of Osteogenic Markers in a Human Osteoblast Cell Line
Source: Cells. 2023 Jan 19;12(3):364. doi: 10.3390/cells12030364 (PMC9913848; doi:10.3390/cells12030364)
Supplement: Supplementary file 1 [file cells-12-00364-s001.zip › cells-2010317-supplementary.pdf]

cells

MDPI

Bacillus subtilis modulated the expression of osteogenic markers in hFOB1.19 cell line

Jerry Maria Sojan [1], Caterina Licini [2], Fabio Marcheggiani [1], Oliana Carnevali [1*], Luca Tiano [1], Monica Mattioli-Belmonte [2+], and Francesca Maradonna [1+*]

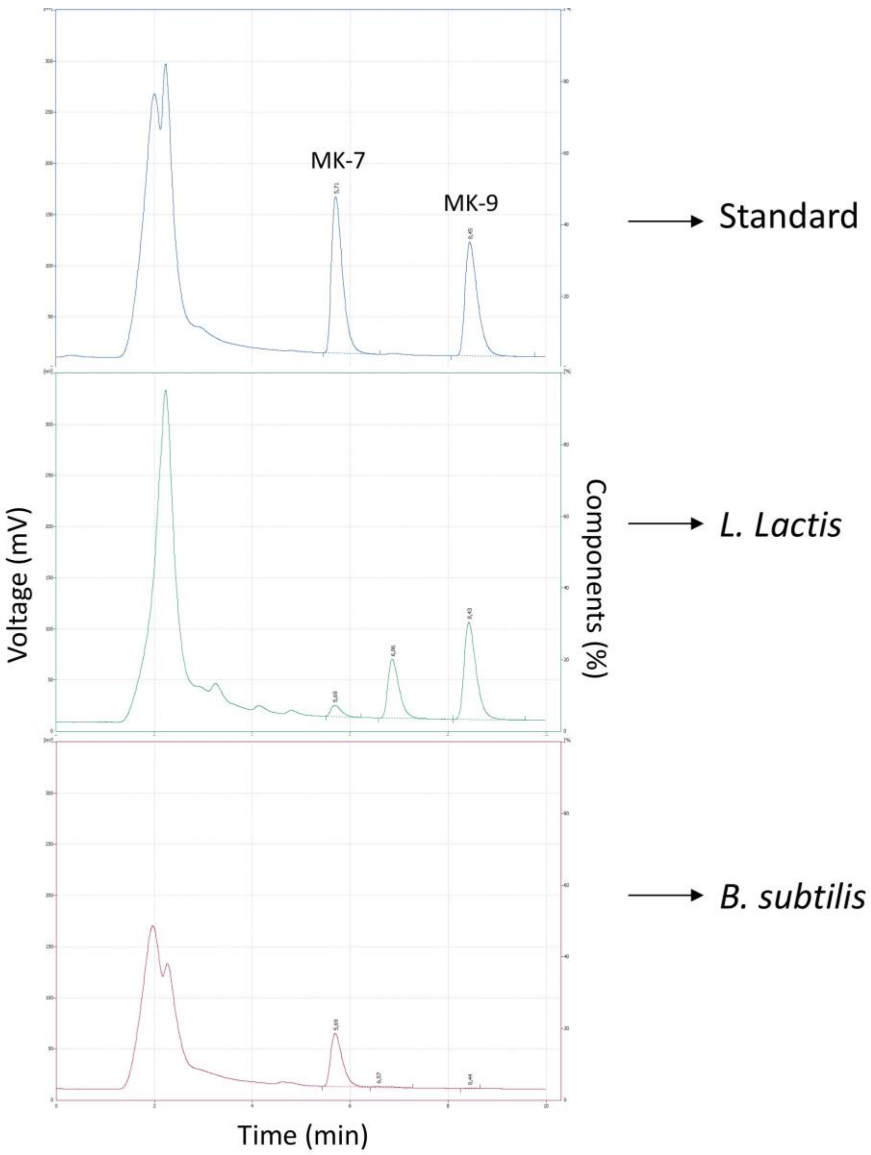

Supplementary Figure S1. MK-7 and MK-9 peaks of the standard and in the two probiotic species observed in the HPLC output.

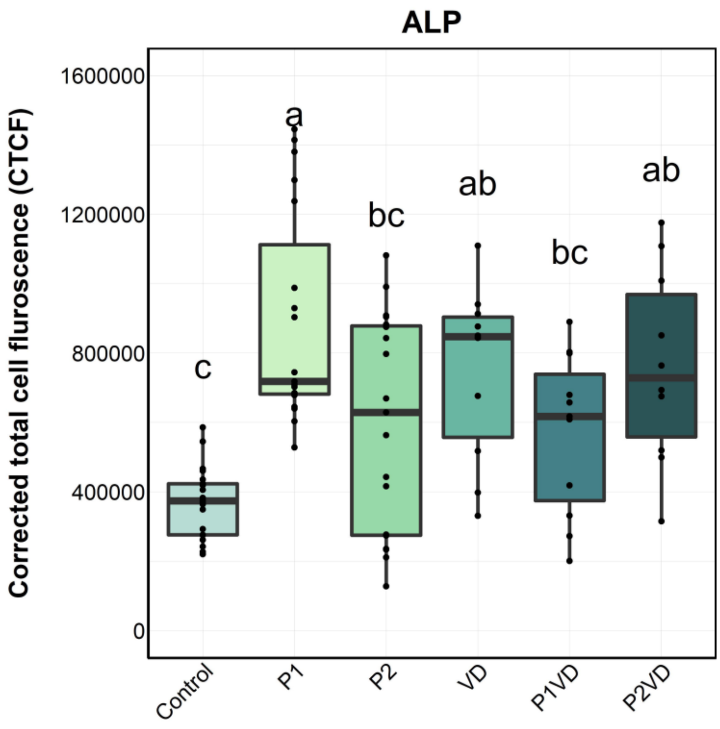

Supplementary Figure S2. Semi-quantitative analysis to calculate corrected total cell fluorescence (CTCF) in hFOB1.19 cells cultured without any treatment (Control), treated with VD (VD), with two probiotics (P1 and P2) and their respective combinations with VD (P1VD and P2VD. One-way ANOVA was used to compare between groups and Tukey's post hoc test was used for multiple comparisons among all the groups. Data are shown as means ± SD as error bars. Different letters above each boxplot denote statistically significant differences among experimental groups (n=20; P <0.05). Three groups that differ significantly from each other are labelled a, b, and c, and two that differ from each other but not from the third are labelled a, b, and ab.
